# Supplementary material for: Comparative performance analysis of low-enthalpy geothermal energy in arid and semi-arid climates
Source: Sci Rep. 2026 May 5;16:14279. doi: 10.1038/s41598-026-47489-4 (PMC13144698; doi:10.1038/s41598-026-47489-4)
Supplement: Supplementary file 1 — Supplementary Material 1 [file 41598_2026_47489_MOESM1_ESM.pdf]

Comparative Performance Analysis of Low-Enthalpy Geothermal Energy in Arid and Semi-Arid Climates

Anwar Hegazy[1,*]

[1]Department of Mechanical Engineering, Arab Academy for Science, Technology and Maritime Transport, Alexandria, 1029, Egypt
[*]anwarhegazy.ah@gmail.com ; anwar.hegazy@aast.edu

ABSTRACT

This study utilizes a model to investigate the thermal performance and determine the optimal design parameters for the low-enthalpy geothermal energy system earth-air heat exchanger in two climatically distinct regions: the arid, high-extreme environment of the Upper Egypt region and the milder conditions of the Egyptian Mediterranean region. First, a parametric analysis was conducted, focusing on the influence of pipe length, installation depth, air velocity, and pipe diameter, using Typical Meteorological Year data. The analysis identifies an optimal design configuration, recommending a pipe length of 40 m–50 m, an air velocity of 2 m/s, and a practical installation depth of 3 m–5 m to maximize heat exchange efficiency without incurring unnecessary costs. Using optimal design conditions, performance comparison reveals that earth-air heat exchanger effectiveness is directly proportional to the magnitude of the ambient-to-soil temperature difference. At peak hours, the temperature drop in the Aswan arid region was found to be 45% higher than in the Alexandria semi-arid region, highlighting the system's enhanced effectiveness in arid environments. Furthermore, the earth-air heat exchanger demonstrates year-round potential by providing effective heating during winter months when the soil is warmer than the ambient air at both regions. It is demonstrated that the low-enthalpy geothermal system hold a higher energy-saving potential in arid regions with extreme temperature conditions.

Introduction

The escalating global energy demand, primarily fueled by continuous population growth and rapid urbanization, presents a profound dual challenge to the environment and the economy in the 21st century[1]. This challenge is acutely felt in regions characterized by extreme arid climates, most notably the Middle East and North Africa (MENA)[2]. Due to high summer temperatures, the MENA region has become heavily dependent on mechanical cooling. Air conditioning (AC) is now indispensable across residential, commercial, and industrial sectors, making it the single largest contributor to the electrical peak load and overall energy consumption in many regional markets[3]. This reliance on energy-intensive conventional cooling not only stresses national power grids but also contributes substantially to greenhouse gas emissions, thus necessitating an urgent transition toward sustainable, low-energy thermal management solutions[4].

Addressing this deep dependency requires a dedicated investigation into alternatives to traditional Vapor Compression Cycle (VCC) systems. While incremental improvements in insulation and appliance efficiency offer some relief, a significant, enduring reduction in the cooling load can only be achieved by implementing passive building technologies[5]. These passive cooling techniques leverage natural energy sinks and heat transfer mechanisms, offering a pathway toward dramatically lowered operational costs and environmental footprints. Among these, Earth-Air Heat Exchanger (EAHE) system is globally recognized for its potential. The EAHE operates by utilizing the thermal inertia of the subsurface soil, which maintains a relatively stable temperature—significantly cooler than the ambient air during the summer and warmer in the winter[6]. The EAHE system involves burying a network of pipes at a sufficient depth to harness this stable temperature profile, allowing ambient air to be circulated through them[7]. This process results in the pre-conditioning of the air—cooling it in the summer and pre-heating it in the winter—before it is delivered to the building's HVAC system or directly into the space[6]. This vital pre-conditioning function dramatically reduces the temperature difference that the mechanical system must overcome, consequently lowering the overall energy consumption and peak load demand of the building's thermal management system[8]. This potential has driven several applications, including building-integrated systems[9], air handling units[10], and methods for enhancing photovoltaic efficiency in arid climates[11].

The efficiency and overall feasibility of an EAHE system are fundamentally governed by several interdependent factors, highlighting a strong location dependency[12]. These critical parameters fall into three main categories: heat exchanger design parameters, the soil subsurface temperature profile, and the soil thermal properties[12]. Investigations have consistently shown that the soil subsurface temperature profile is intricately linked to site-specific environmental factors occurring at the soil

surface, demonstrating a crucial dependence on the local climate[13]. Similarly, the soil thermal properties—including structure, moisture, and texture—significantly influence the efficiency of heat transfer and, consequently, the overall EAHE performance[14]. Specifically, the optimal operating parameters of an EAHE system are dependent on the nature of the soil texture and soil temperature at the relevant installation depth, as highlighted in recent review articles[12]. Investigations have shown that the soil subsurface temperature is tightly linked to the weather at the installation site[7]. It is therefore evident, as recent reviews demonstrated[15], that EAHE performance is tightly linked to the climate condition of interest. This becomes particularly relevant in the MENA region, where the climate is predominantly arid or semi-arid[16]. These two distinct climates will inherently influence and determine the EAHE's performance characteristics. Assessing the thermal properties of the soil at an installation site requires a comprehensive evaluation of soil classification[7]. While heat transfer textbooks provide foundational estimation methods based on geological sampling and textural analysis[14], a robust assessment must account for the site-specific variables that dictate thermal conductivity and resistance. Whereas obtaining accurate data on the soil subsurface temperature is an essential yet challenging requirement. While on-site field measurements provide the most direct and accurate data, they are often prohibitively expensive and logistically demanding when dealing with vast, undeveloped tracts of land[7]. This challenge is acutely felt in regions like Egypt, where the landscape is over 94% desert. Consequently, mathematical modeling has emerged as the accepted standard for estimating these subsurface soil temperatures[17]. This method predicts the soil's thermal behavior by utilizing readily available meteorological data[18]. To ensure reliability, models have been specifically adapted for arid and semi-arid regions to estimate these crucial sub-soil temperatures employing standard data sets either Typical Meteorological Year (TMY) data[18–20] or remotely sensed data[7,17]. Such approach allowed unlocking the potential of low enthalpy geothermal systems like EAHE[7].

Extensive research has already established the viability of EAHE systems within arid and semi-arid climates, with significant contributions focusing on localized studies in Egypt[19], Australia[21], Algeria[22,23], and Saudi Arabia[24]. However, despite this growing body of evidence, the literature lacks a comprehensive comparative analysis that addresses how EAHE performance varies across the diverse geographical and climatic landscape of the MENA region. This study aims to bridge this gap by evaluating EAHE performance dynamics in direct relation to the varying environmental conditions found throughout the area.

To achieve this, the research utilizes a comparative framework involving a semi-arid representative site in North of Egypt and an arid site in the Upper Egypt region, serving as representatives for the broader MENA territory. The methodology employs TMY data integrated with mathematical modeling to accurately predict subsurface soil temperatures. These profiles are then coupled with an EAHE model to simulate thermal exchange efficiency. Furthermore, a detailed parametric sensitivity analysis was conducted to identify optimal design configurations for the heat exchanger. By accounting for the distinct soil textures and thermal properties characteristic of the desert terrains in both locations in Egypt, this study ensures that its findings are specifically tailored to the unique geological and climatic demands of the region.

Methods

Study location

This investigation focuses on two distinct urban environments: Aswan, Egypt, and Alexandria, Egypt. Aswan serves as a representative study location for the hyper-arid regions of the Nile Valley in Upper Egypt. Geographically, it is situated on the east bank of the Nile River, positioned at coordinates approximately 24°05′20″N and 32°53′59″E. Under the Köppen climate classification system, the city falls under the hot desert climate (BWh) classification. It is recognized as one of the driest and hottest inhabited places in Egypt, characterized by consistent year-round aridity and extreme summer temperatures. In contrast, the coastal city of Alexandria provides a representative environment of the unique intersection between the Mediterranean maritime climate and the North African arid zone. Situated along the northern coast of Egypt, the city lies approximately 183 km northwest of Cairo at coordinates around 31°12′0″N and 29°55′0″E. According to the Köppen climate classification system, Alexandria is classified as a hot semi-arid climate (BSh), distinguished from Aswan by its significant maritime influence and concentrated winter rainfall.

System description

The operating principle of an EAHE in hot climates is as follows. A fan draws warm ambient air into a network of pipes buried underground. Since the soil temperature remains lower than the outdoor air, a heat exchange occurs where the air sheds its thermal energy into the surrounding soil. This process cools the air before it is directed into the building's interior. To model that, we first need to know the temperature of the soil at the EAHE installation depth. Then, we model the heat transfer between the air and the surrounding soil.

Soil temperature modelling

The soil temperature variation with depth is considered as a one-dimensional heat conduction through a semi-infinite solid domain. The one dimensional form of the heat equation is given as[25]:

$$\frac{\partial^2 T}{\partial z^2} = \frac{1}{\alpha}\frac{\partial T}{\partial t} \tag{1}$$

The surface boundary is assumed to follow a cyclic temperature variation so the following boundary condition at the surface is considered[25,26]: $T(0,t) = T_{mean} - A_s \times \cos(\omega(t - t_o))$. Theoretically as depth tends to infinity the interior node is subject to the following boundary condition[25]: $T(\infty, t) = T_{mean}$. Accordingly the temperature of the soil can be calculated from[26] where α and z are soil thermal diffusivity and ground depth respectively; noting that in this equation α is in (m^2/day):

$$T(z,t) = T_{mean} - A_s \times e^{-z\sqrt{\frac{\pi}{365\alpha}}} \times \cos\left[\frac{2\pi}{365}\left(t - t_o - \frac{z}{2}\left(\sqrt{\frac{365}{\pi\alpha}}\right)\right)\right] \tag{2}$$

where T_{mean} is mean surface temperature, A_s temperature fluctuation amplitude and z is soil depth.

EAHE modelling

Heat transfer within the pipe of the EAHE occurs through two distinct regimes: first, by convection between the air flowing inside and the pipe's inner wall; and second, by conduction through the pipe inner wall itself and into the surrounding soil. The crucial convection heat transfer component is mathematically calculated using the Nusselt number, following standard fluid dynamics and heat transfer principles[25],

$$h_{convection} = \frac{Nu \times k_a}{d_{p,in}} \tag{3}$$

where k_a is the Air thermal conductivity, $d_{p,in}$ is the inner pipe diameter and the Nusselt number (Nu) is calculated from the following correlations[25] for turbulent flow, $Nu = 0.023Re^{0.8}Pr^n$, where n is 0.3 for cooling and 0.4 for heating, and for laminar flow,$Nu = 3.66$. The Reynolds number (Re) and Prandtl number (Pr) inside the pipe are given by, $Re = \rho v_{air} d/\mu$ and $Pr = c_p \mu / k_{air}$. The EAHE is treated as heat exchanger with constant wall temperature. The NTU-effectiveness method is applied which gives the following equations[25]:

$$\varepsilon = 1 - e^{-NTU} \tag{4}$$

Where the effectiveness (ε) , number of transfer units (NTU) and overall heat transfer coefficient (U) are given by,

$$\varepsilon = \frac{T_{ambient} - T_{out}}{T_{ambient} - T(z,t)} \tag{5}$$

$$NTU = \frac{UA}{\dot{m}c_p} \tag{6}$$

$$\frac{1}{UA} = \left[\frac{1}{2\pi r_i L h_{convection}} + \frac{\ln(\frac{r_e}{r_i})}{2\pi L k_{pipe}}\right] \tag{7}$$

where the area of heat transfer is equal to $A = \pi d_o L$. Accordingly for any axial length of the pipe, the outlet temperature (T_{out}) can be calculated from,

$$T_{out} = T_{ambient} + (T(z,t) - T_{ambient}) \times (1 - e^{-NTU}) \tag{8}$$

The mass flow rate of air is calculated from, $\dot{m} = \frac{d^2}{4}\pi\rho v_{air}$. Considering a one horizontal pipe in an open-loop configuration, the cooling capacity of the EAHE is calculated by the following equation,

$$\dot{Q}_{cooling} = \dot{m}c_p(T_{ambient} - T_{out}) \tag{9}$$

The cooling capacity ($\dot{Q}_{cooling}$) is calculated assuming ($T_{ambient}$) is higher than the soil temperature at the installation depth where an air cooling process occurs. So in that sense, a positive value of cooling capacity indicates that air is undergoing a cooling process and a negative sign indicates it is undergoing a heating process. The research in Hegazy et al.[18] verified the accuracy of the described EAHE model in predicting the air outlet temperature (T_{out}) from a 0.1601 diameter PVC pipe in cooling operation mode by comparing it to experimentally measured data in Ref.[27] for an air velocity range 2-5 m/s, demonstrating a prediction error of only 2.4%. Following that, the influence of that error margin on the cooling capacity ($\dot{Q}_{cooling}$) was calculated using the following equation[7]:

$$\frac{\partial(\dot{Q}_{cooling})}{\partial(T_{out})} = -\dot{m}c_{p,a} \tag{10}$$

The absolute value of the uncertainty in the cooling rate was calculated using the following equation[7]:

$$\delta\dot{Q}_{cooling} = \left|-\dot{m}c_{p,a}\right| \cdot \delta T_{out} \tag{11}$$

The analysis of uncertainties, revealed that the calculated cooling or heating capacity may vary by up to $\pm 0.5W$ which stems from the 2.4% margin of error associated with the EAHE model's predictions[7].

Results and Discussion

Soil subsurface temperature profile

To effectively calculate the performance of the EAHE, specific input data is essential, including the ambient air temperature, the soil thermal properties, and the soil temperature at the intended installation depth. The necessary climatic data for the selected locations, Aswan, and Alexandria, was sourced from the TMY weather file provided in Ref.[28], which compiles hourly averaged data over a representative period of records, typically 30 years. Furthermore, the specific soil properties utilized for the performance calculations for both cities are detailed in Table.(1). The thermal properties of desert sand were sourced from Ref.[29] for the Aswan case and from Ref.[30] for the Alexandria case.

Table 1. Soil thermal properties

City	Aswan	Alexandria
Soil Density	1775	2050
Soil specific heat	840	1840
Soil thermal conductivity	0.91	2.806

To calculate the soil temperature at a specific depth using the governing equation Eq.(2), three key parameters are needed: the ground surface mean temperature (T_{mean}), the amplitude of surface temperature fluctuation (A_s), and the phase constant (t_o). Typically, obtaining the first two values requires long-term surface temperature data, which is often unavailable. However, based on the approach by Watson and Labs[26], the mean surface temperature (T_{mean}) can be approximated by adding 1.7°C to the average annual air temperature. Similarly, the annual temperature fluctuation amplitude (A_s) from the mean surface temperature can be estimated by adding 1.1°C to one-half the difference between the July and January monthly average air temperatures. Based on the analysis of the TMY data for the selected locations, the calculated values for T_{mean} and A_s were 27.30°C and 13.60°C for Aswan, and 22.08°C and 6.75°C for Alexandria, respectively. Regarding the phase constant (t_o), it is determined using the periodic heat-conduction theory, which posits that the phase of solar radiation lags behind the cyclic wave of surface temperature by 1/8 of a cycle, or 46 days. Since the day of minimum solar radiation occurs on day 355 of the year, counting 46 days forward from that point yields a value for t_o of 36 (representing day 36 of the subsequent year).

The temperature profile, as illustrated in Fig. (1a) for Alexandria and Fig. (1b) for Aswan, exhibits a sine wave variation over time. A key observation is that the amplitude of this fluctuation markedly decreases with increasing depth. This phenomenon also creates a lag in the temperature amplitude, meaning that at the same point in the year, the temperature of the deeper ground layers is significantly more stable than that of the upper surface. Importantly, at a depth of 4 meters and below, the ground temperature variation is minimal year-round. This stability makes the deep ground a highly reliable heat sink/source, which is defined as the earth's undisturbed temperature.

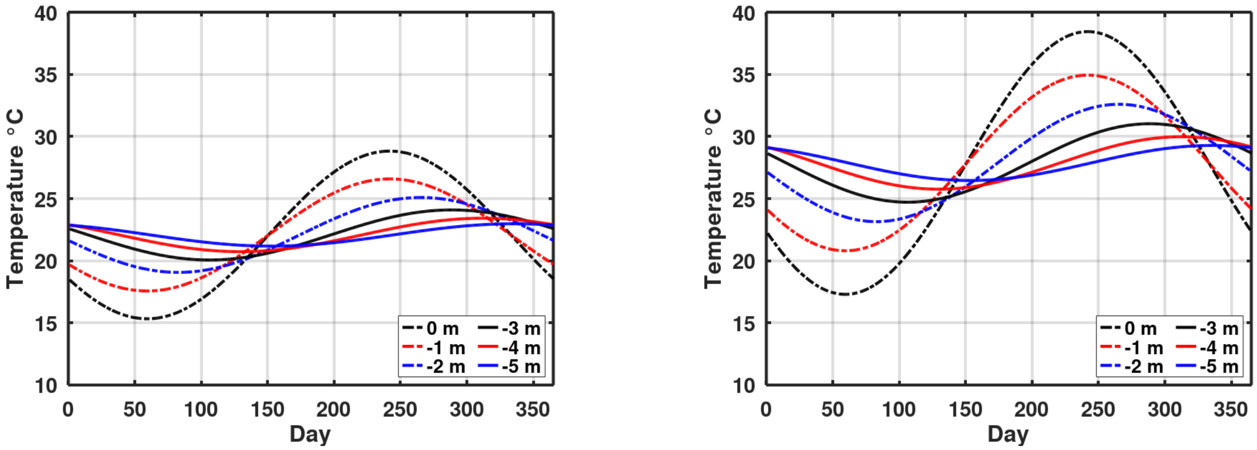

(a) Alexandria **(b)** Aswan

Figure 1. Ground Temperature variation with depth

EAHE pipe parametric analysis

A parametric analysis was conducted to investigate the influence of key design variables—pipe length, installation depth, and air velocity—on the EAHE performance. This analysis and the subsequent performance comparison were executed for the operating conditions of Aswan and Alexandria, specifically focusing on the warmest hour of the year at both sites. For Aswan, this peak occurred in August with a notably high temperature of 40.7°C, whereas for Alexandria, the warmest hour in August had a lower temperature of 30.1°C. Beyond this peak-hour analysis, hourly monthly averaged temperature values were also utilized to monitor the EAHE's performance throughout an entire annual cycle. The pipe material of choice was PVC, based on its reliability and rust resistance. The specific material properties of the PVC pipe used in the EAHE design for these calculations are[7]: PVC thermal conductivity = 0.161 (W/m.K); PVC specific heat = 900 (J/kg.K); PVC density = 1380 (kg/m^3); Pipe wall thickness = 0.005 (m).

Fig. (2a) illustrates the variation of air temperature along the pipe length at various installation depths in Aswan. A significant temperature drop is observed within the first 20 meters of pipe length. Beyond this point, the temperature continues to decrease gradually until it begins to flatten out at approximately 35 meters, becoming nearly constant in the range of 40–45 meters. Moreover, the analysis revealed that increasing the installation depth of the EAHE leads to a greater temperature difference between the inlet and outlet air, but this performance improvement is only notable up to a depth of 4 to 5 meters. Beyond this range, there is no substantial improvement in the temperature differential. Consequently, installing the EAHE deeper than 5 meters results in unnecessary excavation expenses without providing any significant performance advantages. For example, the temperature drops recorded at 1 meter, 3 meters, 4 meters, and 5 meters, were 5.7°C, 9.2°C, 11.1°C, and 11.4°C, respectively, highlighting the diminishing returns past the 5-meter mark.

Fig. (2b) illustrates the impact of air velocity variation on the air temperature along the pipe length. It can be deduced that as the air velocity increases, the rate of temperature decrease along the pipe is affected, evidenced by the steepness of the curves. Importantly, the final air temperatures at the end of the pipe (between 40 and 45 meters) are approximately equal regardless of the initial velocity. This convergence occurs because the air temperature approaches the pipe wall temperature as the length increases, eventually reaching a point (around 40–45 meters) where the air temperature is equal to the pipe wall temperature, causing no further significant heat exchange with the surrounding soil. Since the air flow rate is directly proportional to air velocity, a higher velocity necessitates a longer contact distance for the air to reach this equilibrium wall temperature, a behavior clearly shown in Fig. (2b). Nonetheless, a pipe length of 40–50 meters proves sufficient for air velocities between 2 m/s and 5 m/s to achieve the pipe wall temperature. It is note worthy that higher velocities lead to higher fan power consumption and increased friction between the air and the pipe's inner wall, potentially causing an air temperature rise—factors that were not accounted for in this specific parametric study. Accordingly, the 2 m/s velocity was chosen for the simulation as it yields a better thermal performance compared to higher velocities, as visually demonstrated in Fig. (2b).

Fig. (2c) illustrates the effect of changing the pipe diameter on the air temperature along the pipe length, using a fixed air velocity of 2 m/s. It is concluded that the change in pipe diameter has a significant influence on the air temperature, primarily because the air mass flow rate is directly proportional to the square of the pipe diameter, as demonstrated in Eq. (8). As the graph shows, an increase in pipe diameter leads to a corresponding increase in the final air temperature, a result that is particularly significant for diameters between 0.2 m and 0.5 m. For smaller diameters, specifically between 0.1 m and 0.2

m, the effect on air temperature is apparent for pipe lengths lower than 40 m, while for pipe lengths greater than 40 m, the difference between the inlet and outlet temperatures becomes relatively insignificant.

Based on the parametric analysis conducted, the following optimal parameters are recommended for the EAHE pipe design :

- A pipe installation depth of 5 m yields the best temperature drop performance, with negligible further improvement beyond this depth. However, when factoring in digging costs, a depth between 3 m and 4 m is generally recommended as a more cost-effective compromise.

- The recommended pipe length to ensure the air temperature reaches equilibrium with the soil temperature is 40 m to 50 m.

- Diameters between 0.1 m and 0.2 m provide the highest temperature drop. If a higher flow rate is required, it is strongly advisable to utilize multiple pipes in parallel rather than simply increasing the diameter of a single pipe, which reduces cooling efficiency.

- Within the studied range of air flow velocity (2 m/s to 5 m/s), no significant difference in temperature drop was observed for pipe lengths exceeding 40 m, suggesting 2 m/s is optimal due to lower fan power requirements.

Arid and semi-arid EAHE performance comparison

Based on the preceding parametric analysis, the optimal parameters utilized to simulate the EAHE performance in Aswan and Alexandria are: Pipe diameter = 0.1016 m; Pipe length = 50 m; Pipe installation depth = 4 m; Air velocity = 2 m/s. Fig. (3) presents a crucial comparison of the air temperatures along the length of the pipe during the warmest hour of the year at both locations. The resulting temperature difference between the inlet (ambient) and outlet temperatures was 7.59°C for Alexandria and 11.1°C for Aswan. In terms of thermal reduction, the Aswan arid region demonstrated a 45% greater temperature drop compared to the results observed in the semi-arid Alexandria region. This difference strongly suggests that the EAHE performance is more effective and beneficial when the ambient temperature is higher.

Although the system shows high efficiency during warm weather, this does not imply that its relative performance is superior in cooling modes compared to heating modes. Fig. (4) and Fig. (5) plot the monthly hour averaged ambient temperatures alongside the outlet temperatures for each month. A crucial observation is made during the winter months: the ambient temperature is lower than the EAHE outlet temperature. This inversion occurs because the soil temperature at the installation depth remains warmer than the ambient air, allowing the EAHE to pre-heat the incoming air rather than cool it. During the winter months, particularly in Alexandria, the temperature difference between the ambient air and the soil is actually bigger than the difference observed during the summer months. Consequently, the EAHE's potential is more effective in winter in Alexandria for providing heating, as evidenced by the higher absolute value of the temperature potential shown in Fig. (6) and Fig. (7). Therefore, it is concluded that the potential of the EAHE should be assessed based on the absolute temperature difference between the inlet air and the soil temperature at the installation depth, irrespective of the season (i.e., whether the unit is cooling or heating). Overall, the results indicate that the EAHE has a higher performance potential in arid regions with high temperature extremes, as exemplified by Aswan, compared to regions with less extreme, milder ambient temperatures, such as Alexandria.

Study limitations and Future work

While the initial findings demonstrate the fundamental performance of the EAHE system, certain simplifications were adopted that outline clear directions for future research. A primary limitation lies in the treatment of soil properties for the Aswan and Alexandria study sites. In the current model, these properties were treated as localized aggregate values, making it difficult to decouple the specific influence of local climate conditions from the inherent thermal characteristics of the soil. To better understand which factor exerts a more significant influence, future investigations should employ a detailed sensitivity analysis, potentially within a controlled lab-scale environment, to isolate these variables.

The physical configuration of the system also presents opportunities for expansion. This study considered a single, smooth PVC horizontal pipe; given this simple geometry, the fan power required to circulate air was calculated at an insignificant 0.8 Watt[18]. However, transitioning to more complex pipe networks—incorporating various materials, bends, and elbows—would introduce higher friction and pressure drops, necessitating a more rigorous consideration of fan power and its impact on overall efficiency. Furthermore, while the current use of a 50-meter pipe length mitigates the sensitivity of the outlet temperature to localized thermal resistance by allowing the fluid to approach the soil temperature[18], it does not fully account for the transient effects of soil thermal saturation over extended operation.

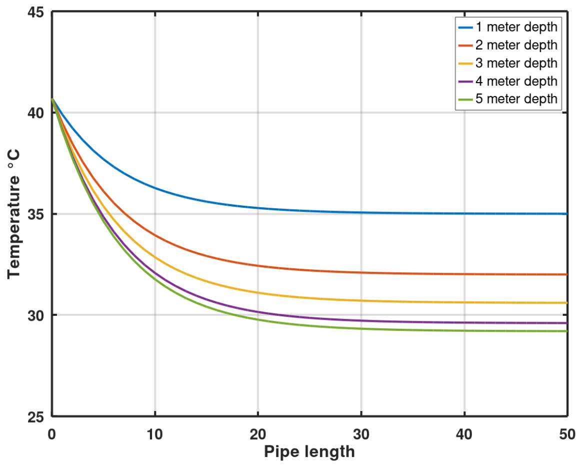

(a) Different installation depths

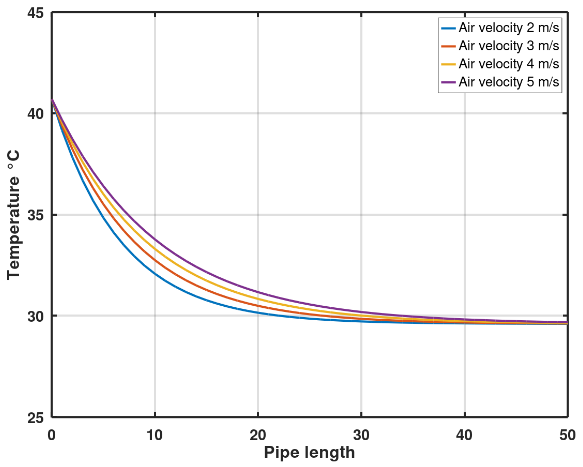

(b) Different air velocities

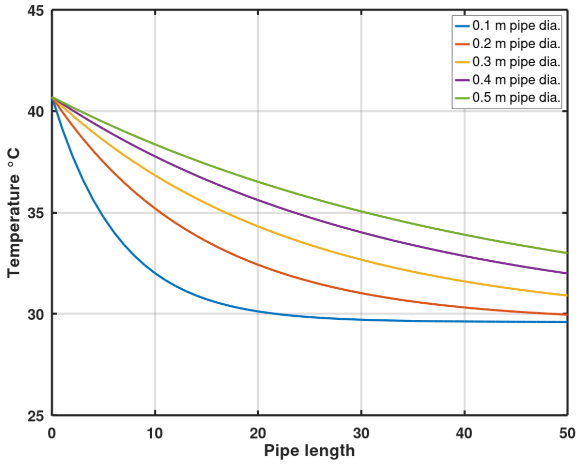

(c) Different pipe diameters

Figure 2. Air temperature variation along pipe length, Aswan

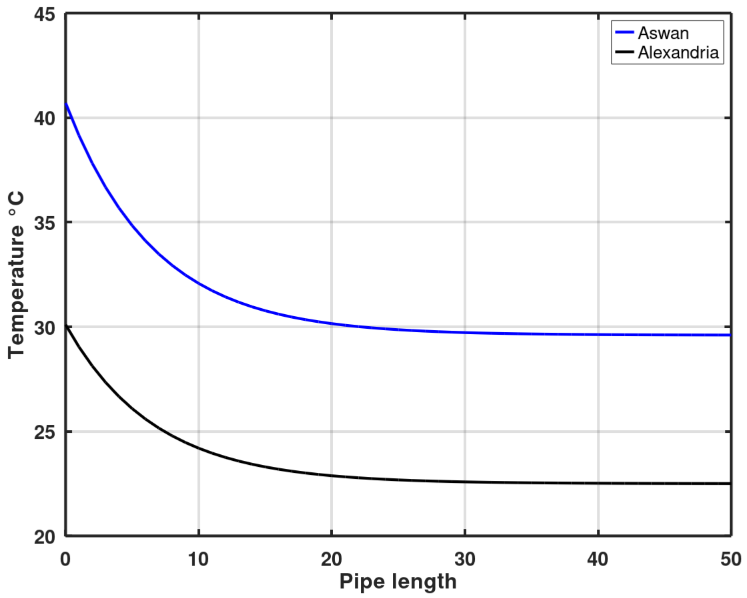

Figure 3. Comparison between air temperature along pipe length in Aswan and Alexandria, Egypt at a depth of 4 m on the warmest hour of the year at both locations

To address these gaps, future research should move toward a comprehensive energy analysis that couples the EAHE system directly to a functional building. Such a setup would allow for a precise evaluation of the Coefficient of Performance (COP) and net energy savings within a real-world climate-control context. By focusing on continuous operation and the integration of diverse pipe materials and geometries, subsequent studies can provide a more robust framework for the large-scale application of geothermal cooling in arid regions.

Conclusion

This study evaluated the performance of an EAHE in the diverse climatic conditions of Aswan and Alexandria, Egypt. The results demonstrate that soil temperature stability increases significantly with depth, with fluctuations becoming minimal at 4 meters and below, establishing this as the optimal installation depth for a reliable heat sink or source. Parametric analysis revealed that while increasing pipe length and installation depth enhances thermal performance, there are diminishing returns beyond a length of 50 meters and a depth of 5 meters. Furthermore, air velocities of 2 m/s and pipe diameter between 0.1-0.2 meters diameter were found to maximize the temperature differential by increasing the contact time and optimizing the air mass flow rate relative to the pipe surface area.

The comparative analysis between the two regions highlights that EAHE systems are particularly effective in arid climates with high temperature extremes. In Aswan, the system achieved a temperature reduction of 11.1°C during peak summer hours—a 45% greater drop than that observed in the semi-arid climate of Alexandria. However, the system also proved highly effective for winter pre-heating, especially in Alexandria, where the temperature potential in winter exceeded that of the summer cooling mode. Ultimately, the effectiveness of the EAHE is dictated by the absolute temperature difference between the ambient air and the undisturbed soil. These findings suggest that EAHE technology is a robust, sustainable solution for reducing building energy loads in Egypt, with the highest efficiency gains realized in regions experiencing significant thermal gradients.

Data availability

The data sets used and analyzed during the current study are available from the corresponding author upon reasonable request.

References

1. Sheng, P., He, Y. & Guo, X. The impact of urbanization on energy consumption and efficiency. *Energy & Environ.* **28**, 673–686 (2017).

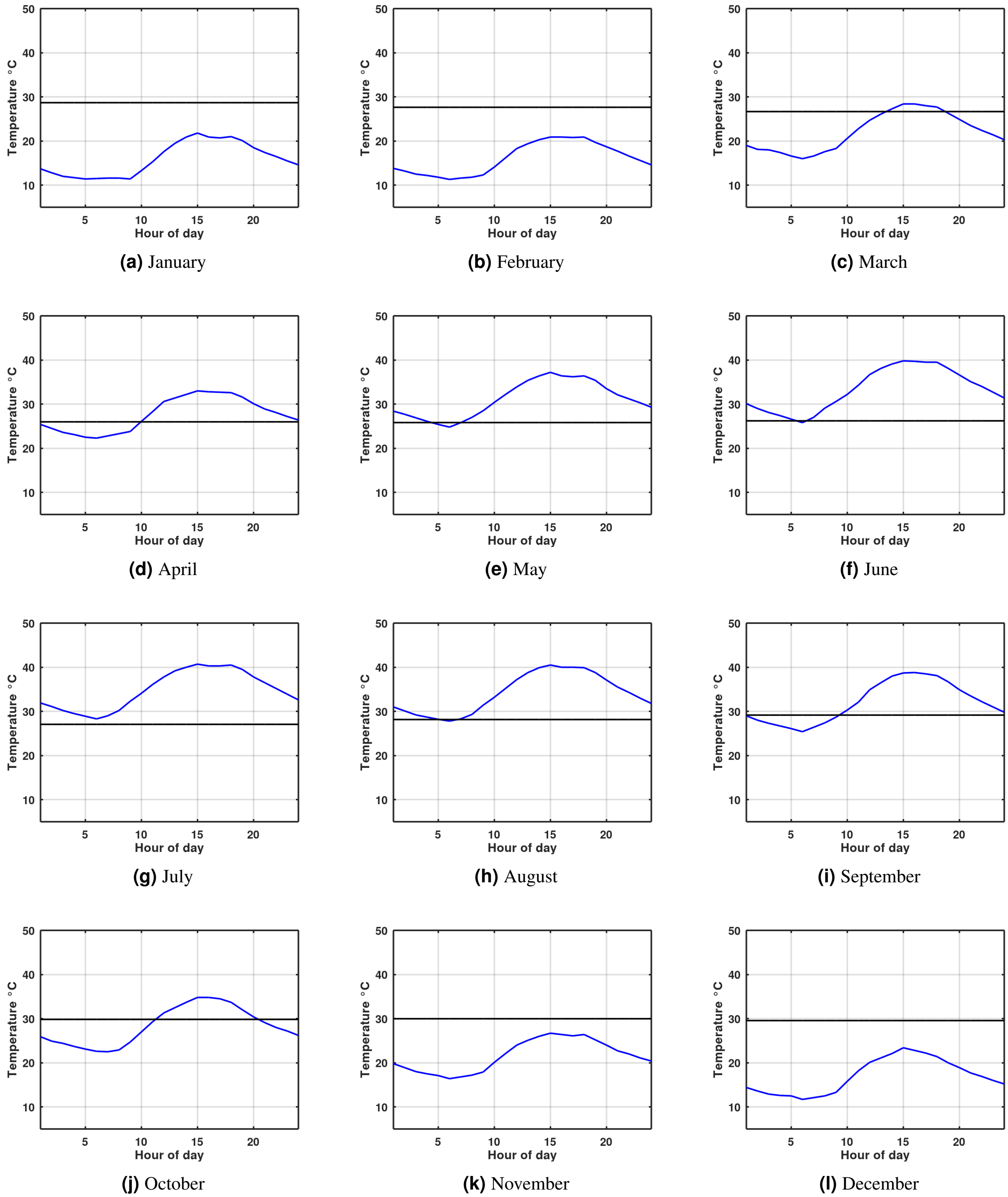

Figure 4. Representative day of each month hourly averaged values of ambient temperature (blue line) against EAHE outlet air temperature (black line) in Aswan for depth of 4 m, pipe diameter 0.1016 m, length of 50 m and air velocity 2 m/s.

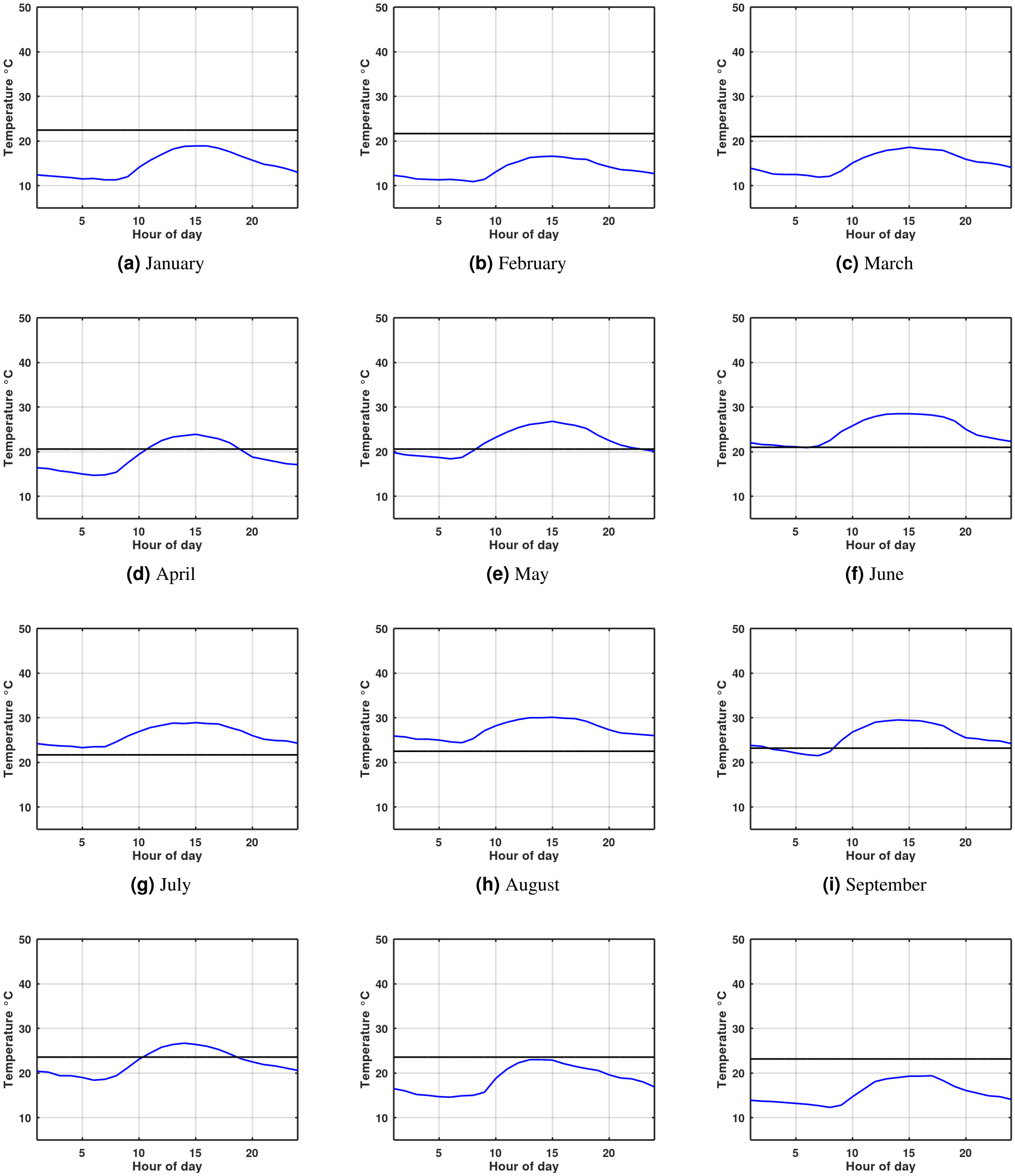

(a) January (b) February (c) March (d) April (e) May (f) June (g) July (h) August (i) September (j) October (k) November (l) December

Figure 5. Representative day of each month hourly averaged values of ambient temperature (blue line) against EAHE outlet air temperature (black line) in Alexandria, Egypt; for depth of 4 m, pipe diameter 0.1016 m, length of 50 m and air velocity 2 m/s

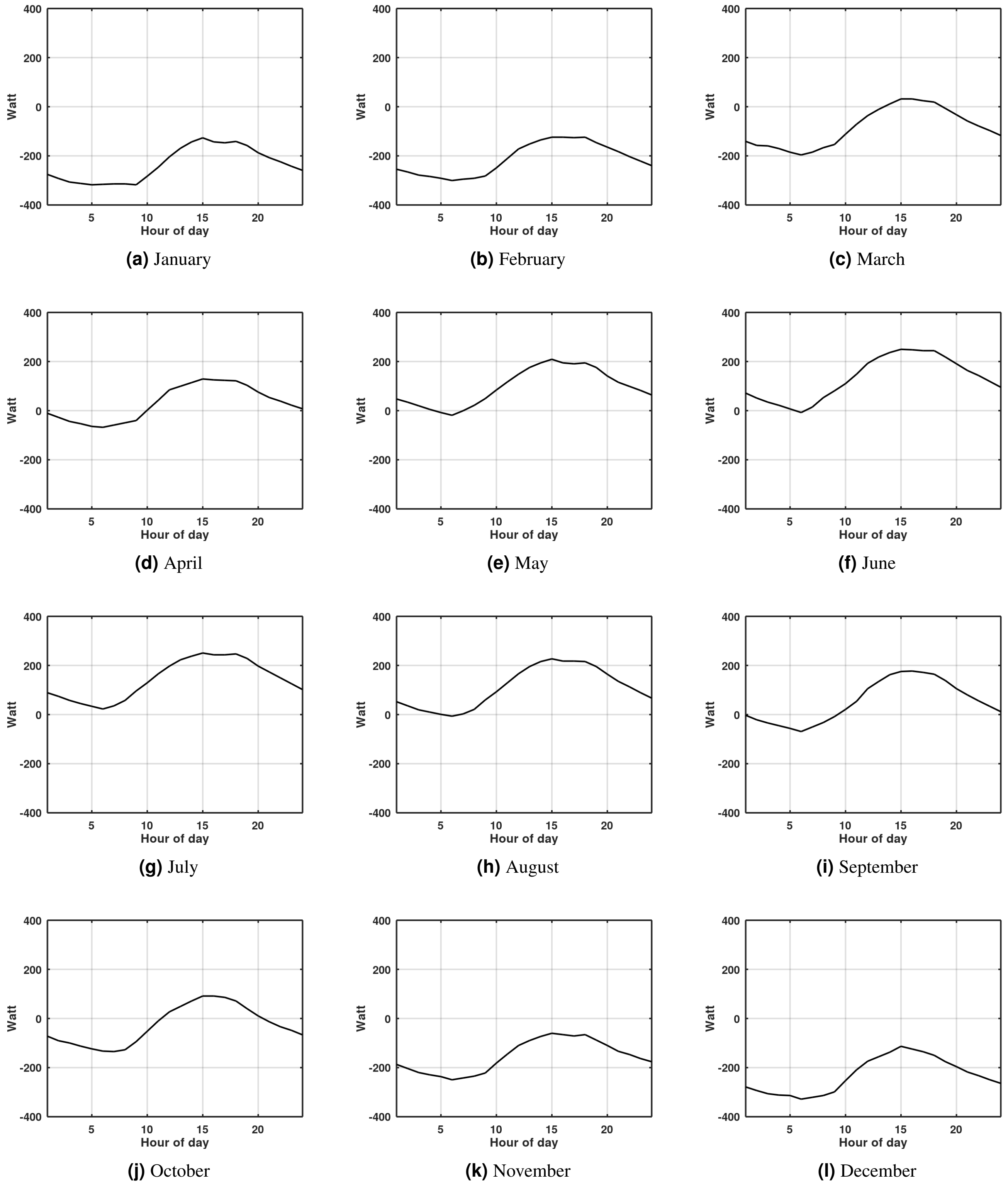

Figure 6. Representative day of each month hourly cooling potential in Aswan, Egypt; for depth of 4 m, pipe diameter 0.1016 m, length of 50 m and air velocity 2 m/s

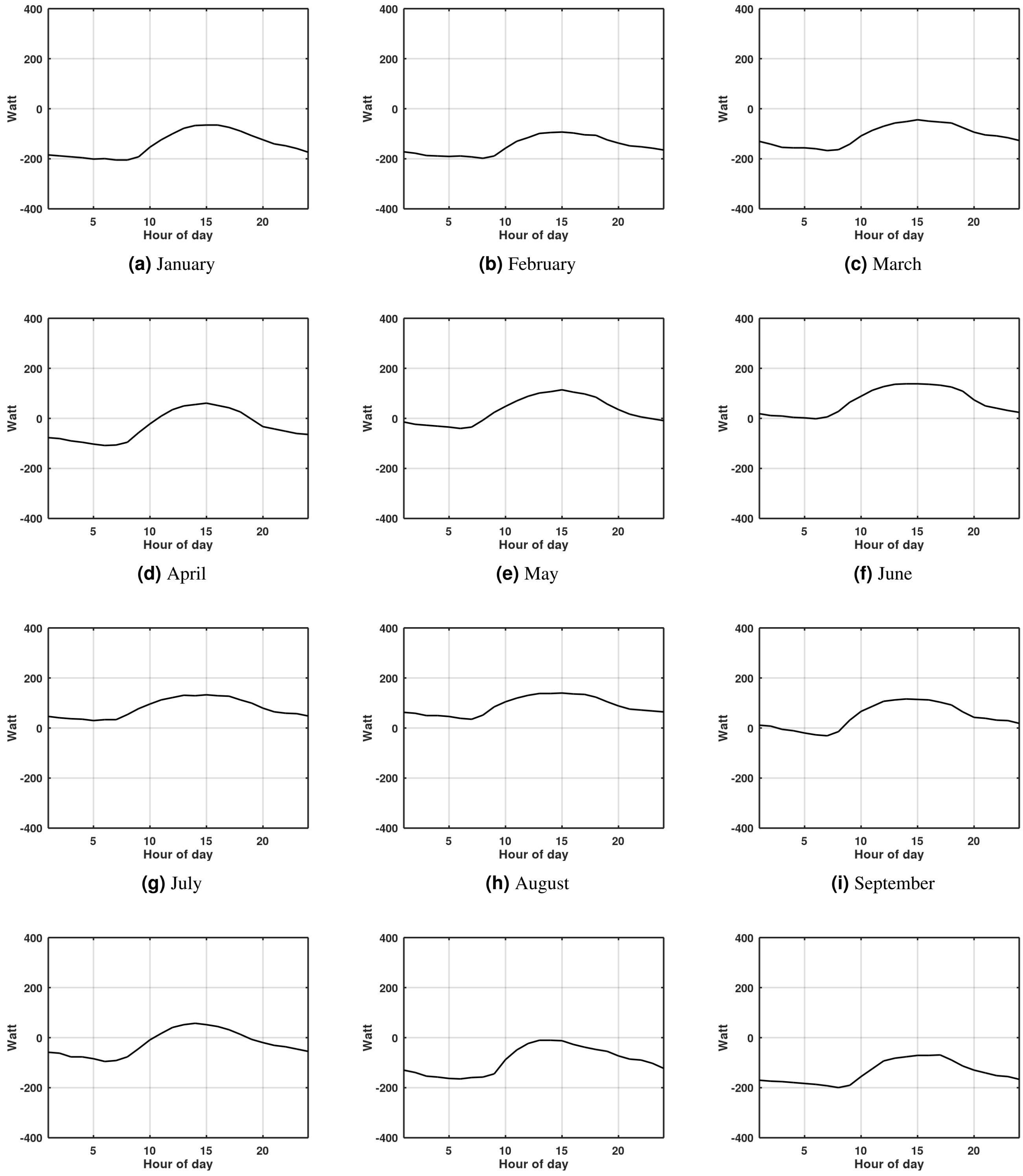

(a) January **(b)** February **(c)** March

(d) April **(e)** May **(f)** June

(g) July **(h)** August **(i)** September

(j) October **(k)** November **(l)** December

Figure 7. Representative day of each month hourly cooling potential in Alexandria for depth of 4 m, pipe diameter 0.1016 m, length of 50 m and air velocity 2 m/s

2. Iyakaremye, V. *et al.* Increased high-temperature extremes and associated population exposure in africa by the mid-21st century. *Sci. Total. Environ.* **790**, 148162 (2021).

3. Howarth, N. *et al.* Staying cool in a warming climate: temperature, electricity and air conditioning in saudi arabia. *Climate* **8**, 4 (2020).

4. Sudarsan, A. & Chithra, K. The role of energy management in transition towards green cities: a review. *Environ. Dev. Sustain.* 1–23 (2024).

5. Sadineni, S. B., Madala, S. & Boehm, R. F. Passive building energy savings: A review of building envelope components. *Renew. sustainable energy reviews* **15**, 3617–3631 (2011).

6. Koshlak, H. A review of earth-air heat exchangers: From fundamental principles to hybrid systems with renewable energy integration. *Energies* **18**, 1017 (2025).

7. Hegazy, A. & Mohamed, S. Z. Unlocking geothermal energy for sustainable greenhouse farming in arid regions: a remote-sensed assessment in egypt's new delta. *Sci. Reports* **13**, 22043 (2023).

8. Mihalakakou, G. *et al.* Applications of earth-to-air heat exchangers: A holistic review. *Renew. Sustain. Energy Rev.* **155**, 111921 (2022).

9. Ali, N. B. *et al.* Synergizing building-integrated photovoltaic with ground-air and water-air heat exchangers for solar-powered gym cooling. *Sci. Reports* (2025).

10. Ali, N. B. *et al.* Performance and cost-benefit analysis of an integrated earth-air heat exchanger and air handling unit system. *Sci. Reports* (2025).

11. Mahmood, D. M. *et al.* Experimental evaluation of a hybrid evaporative and groundwater cooling system for enhancing photovoltaic efficiency in arid climates. *Sci. Reports* **15**, 35571 (2025).

12. Kouki, N., D'Agostino, D. & Vityi, A. Properties of earth-to-air heat exchangers (eahe): Insights and perspectives based on system performance. *Energies* **18**, 1759 (2025).

13. Wu, L. & Zhang, J. Strong subsurface soil temperature feedbacks on summer climate variability over the arid/semi-arid regions of east asia. *Atmospheric Sci. Lett.* **15**, 307–313 (2014).

14. Donde, S. & Maurya, R. Characterizing soil's thermal property for earth air heat exchanger through experiment. *Mater. Today: Proc.* **51**, 1516–1520 (2022).

15. Zhao, J., Huang, B., Li, Y. & Zhao, Y. Comprehensive review on climatic feasibility and economic benefits of earth-to-air heat exchanger (eahe) systems. *Sustain. Energy Technol. Assessments* **68**, 103862 (2024).

16. Hamed, M. M., Sobh, M. T., Ali, Z., Nashwan, M. S. & Shahid, S. Aridity shifts in the mena region under the paris agreement climate change scenarios. *Glob. Planet. Chang.* **238**, 104483 (2024).

17. Hegazy, A. & Govind, A. Assessing the effectiveness of low-enthalpy geothermal energy for greenhouse temperature regulation towards enhancing desert agriculture. *Sci. Reports* **15**, 39014 (2025).

18. Hegazy, A., Farid, M., Subiantoro, A. & Norris, S. Sustainable cooling strategies to minimize water consumption in a greenhouse in a hot arid region. *Agric. Water Manag.* **274**, 107960 (2022).

19. Hegazy, A., Subiantoro, A. & Norris, S. Closed greenhouse heating in an arid egyptian winter using earth-air heat exchangers. In *ASME International Mechanical Engineering Congress and Exposition*, vol. 85673, V011T11A077 (American Society of Mechanical Engineers, 2021).

20. Hegazy, A., Subiantoro, A. & Norris, S. Performance assessment of a closed greenhouse in a hot arid australian climate. In *ASME International Mechanical Engineering Congress and Exposition*, vol. 85673, V011T11A047 (American Society of Mechanical Engineers, 2021).

21. Hegazy, A., Subiantoro, A. & Norris, S. Performance assessment of a closed greenhouse in a hot arid australian climate. In *ASME International Mechanical Engineering Congress and Exposition*, vol. 85673, V011T11A047 (American Society of Mechanical Engineers, 2021).

22. Hamdane, S., Pires, L. C. C., Silva, P. D. & Gaspar, P. D. Evaluating the thermal performance and environmental impact of agricultural greenhouses using earth-to-air heat exchanger: An experimental study. *Appl. Sci.* **13**, 1119 (2023).

23. Cherrad, I., Dokkar, B., Khenfer, N., Benoumhani, S. & Benzid, M. C. Cooling improvement of an agricultural greenhouse using geothermal energy in a desert climate. *Int. J. Energy Environ. Eng.* 1–18 (2022).

24. Al-Helal, I. *et al.* Geothermal energy potential for cooling/heating greenhouses in hot arid regions. *Atmosphere* **13**, 105 (2022).

25. Incropera, F. P., DeWitt, D. P., Bergman, T. L., Lavine, A. S. *et al. Fundamentals of heat and mass transfer*, vol. 6 (Wiley New York, 1996).

26. Watson & Labs. Climatic design: Energy efficient building principles and practices (1983).

27. Bisoniya, T. S., Kumar, A. & Baredar, P. Energy metrics of earth–air heat exchanger system for hot and dry climatic conditions of india. *Energy Build.* **86**, 214–221 (2015).

28. EnergyPlus. Weather Data | EnergyPlus (2019).

29. Al-Sanad, H. & Ismael, N. Thermal properties of desert sands in kuwait. *JOURNAL-UNIVERSITY OF KUWAIT SCIENCE* **19**, 207–207 (1992).

30. Serageldin, A. A., Abdelrahman, A. K. & Ookawara, S. Earth-air heat exchanger thermal performance in egyptian conditions: Experimental results, mathematical model, and computational fluid dynamics simulation. *Energy Convers. management* **122**, 25–38 (2016).

Funding

This research did not receive funding. Open access funding provided by The Science, Technology & Innovation Funding Authority (STDF) in cooperation with The Egyptian Knowledge Bank (EKB).
